# A Great Masquerader: Nasal Type, Extranodal Natural Killer/T-cell Lymphoma Presenting as Recalcitrant Bacterial Sinusitis and Periorbital Cellulitis

**DOI:** 10.1155/2020/6646693

**Published:** 2020-12-15

**Authors:** Zaw Min, Nitin Bhanot

**Affiliations:** Division of Infectious Disease, Allegheny General Hospital, Allegheny Health Network, 420 East North Avenue, East Wing, Suite 407, Pittsburgh, PA 15212, USA

## Abstract

Nasal extranodal natural killer/T-cell lymphoma (ENKL) is a rare clinical entity. It may, however, masquerade as a commonly encountered disease, such as sinusitis. A high index of clinical suspicion of nasal ENKL should be raised when there is inadequate clinical response despite appropriate therapeutic intervention of sinusitis. Biopsy would be warranted and crucial in those instances to make an accurate and timely diagnosis.

## 1. Introduction

Extranodal natural killer/T-cell lymphoma (ENKL), nasal type, is a rare non-Hodgkin's lymphoma and was previously known as lethal midline granuloma or angiocentric lymphoma [1]. Sinusitis is a common disease and has been seen daily in clinical practice. ENKL is an aggressive lymphoma with poor outcome if delayed in diagnosis and therapy [1, 2]. When a patient with ENKL presents with persistent sinusitis or periorbital cellulitis, it may well be a missed diagnosis. A tissue biopsy for pathology is vital to reach an early diagnosis in conjunction with a heightened clinical suspicion and multidisciplinary approach to optimize clinical outcomes.

## 2. Case Presentation

**Figure 1:**
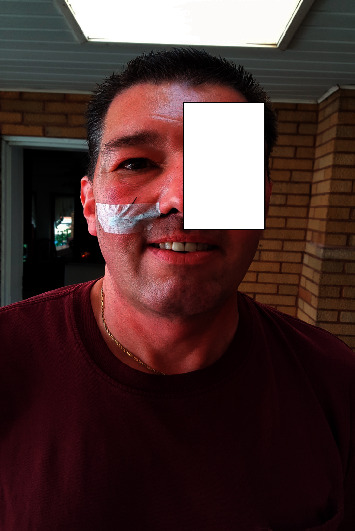
Reduction of swelling and erythema was noted around the right paranasal sinuses and right eye after nasal washout.

A 52-year-old Caucasian male presented to his primary-care physician with a 2-month history of right-sided nasal congestion, fever, and purulent rhinorrhea. His past medical history was significant for gout and seasonal rhinitis. He denied intranasal cocaine use. Despite appropriate courses of oral antibiotics, the patient's symptoms progressed to headache and redness around the right eye. He was referred to an otorhinolaryngologist at that time. Physical examination revealed erythema and edema of the right periorbital tissues, upper and lower eyelids, and cheeks. On palpation, there was no fluctuant mass or exquisite tenderness over the right periorbital region or sinuses. Pupils, extraocular movements, and vision were all intact. The intranasal examination showed considerable swelling of the right middle meatus with purulent drainage. The computed tomography (CT) scan of sinuses showed 100% opacification of the right frontal and ethmoid sinuses, 50% opacification of the right maxillary sinus, and 20% opacification of the right sphenoidal sinus. There was no evidence of orbital or periorbital abscess or bony destruction. Routine laboratory workup was unremarkable. He was admitted to an outside hospital and treated with intravenous vancomycin and cefepime for presumed bacterial sinusitis. Functional endoscopic sinus surgery (FESS) was performed; the operative findings revealed thick yellow purulence and frontal/ethmoidal sinus abscesses. Intraoperative cultures revealed methicillin-sensitive *Staphylococcus aureus* (MSSA). Postoperatively, the patient felt better and the swelling and erythema around the right eye markedly decreased (see Figure 1). The patient was discharged to complete a 2-week course of oral cephalexin for MSSA bacterial sinusitis.

However, 5 days into the oral antibiotic therapy, he experienced worsening of symptoms and was admitted to our institution. He was noted to have marked right periorbital edema with inability to completely open his right eye. Nasal washout on the right was performed. Thick mucinous discharge was noted. No specimens were taken for culture or pathology because it was thought to be severe MSSA bacterial sinusitis that failed outpatient oral antibiotic treatment. The patient was then discharged home with intravenous nafcillin for 2 weeks.

Despite intravenous antibiotic therapy, the patient reported worsening right eye swelling and was readmitted 6 days into his course of intravenous antibiotics. The patient could not open his right eye due to extensive eyelid swelling. Marked periorbital erythema and extensive conjunctival chemosis were noted.

**Figure 2:**
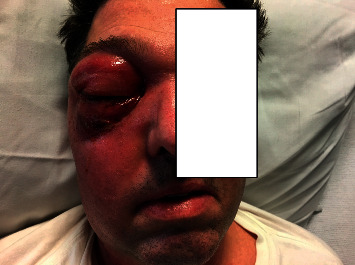
Exuberant periorbital edema, erythema, and superficial excoriation with serous drainage were noted over the right eye and right paranasal region.

MRI of the right orbit with IV gadolinium showed extensive fluid collection in the right frontal and ethmoidal sinuses and soft tissue swelling within the right orbit with right eye proptosis. MRI brain and MRI venogram with IV gadolinium failed to show intracranial extension or cerebral venous sinus thrombosis. The patient underwent an additional FESS of the right frontal and ethmoidal sinuses to optimize source control of the infection. There was no endoscopic finding suggestive of invasive fungal sinusitis. Cultures and biopsy from the sinus tissue were obtained this time. The former revealed the growth of *Enterobacter cloacae* for which ciprofloxacin was added to the antibiotic regimen. Fungal sinus tissue culture was negative. However, the patient continued to have worsening right periorbital edema, erythema, and chemosis despite on appropriate antibiotic therapy (see Figure 2).

Antineutrophil cytoplasmic antibodies test (ANCA) was ordered to rule out ANCA-associated vasculitis or granulomatosis with polyangiitis, and it returned negative. Ophthalmology also evaluated the patient and their examination revealed a normal fundus with no optic nerve edema or venous congestion. The patient had the right anterior orbitotomy and dacryocystorhinostomy for better lacrimal drainage to alleviate periorbital swelling. Right orbital tissue biopsy for pathology was also obtained.

**Figure 3:**
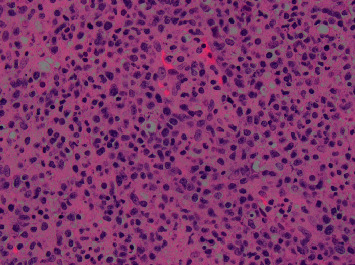
There were dense atypical lymphoid cells with irregular nucleoli (H&E stain, 400X).

**Figure 4:**
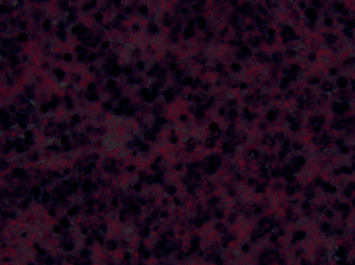
In situ hybridization was diffusely positive for Epstein–Barr virus (EBV) RNA.

The right sinus as well as the orbital tissue pathology illustrated dense small atypical T cells with irregular nucleoli (see Figure 3), which were positive for natural killer cells or cytotoxic T-cell markers—CD2, CD3, and CD56 on the immunohistochemistry study. The neoplastic cells were negative for CD4, CD8, and CD7 on immunophenotype evaluation. Molecular detection of a clonal T-cell receptor (TCR) rearrangement was positive, confirming T-cell origin monoclonal expansion. In situ hybridization was strongly positive for Epstein–Barr virus (EBV) RNA (see Figure 4). Pathologic findings were suggestive of nasal type, extranodal natural killer/T-cell lymphoma. Blood EBV PCR was positive at 3,100 copies/mL (reference, <100 copies/mL). There was no lymphadenopathy or hepatosplenomegaly on CT chest, abdomen, and pelvis as well as no evidence of lymphoma in the bone marrow aspirate and biopsy. All findings were consistent with extranodal natural killer (NK)/T-cell lymphoma, nasal type.

**Figure 5:**
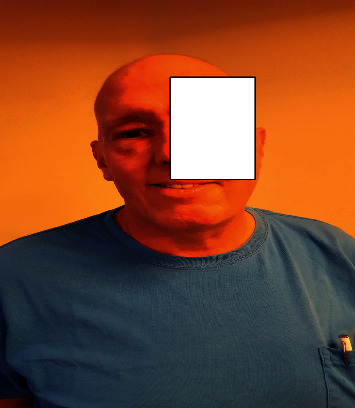
Significant resolution of the right periorbital edema and erythema was noted after 3 cycles of chemotherapy. The right eye was eventually able to fully open.

The patient had completed 4 cycles of SMILE chemotherapy regimen (*S*teroids, *M*ethotrexate, *I*fosfamide, *L*-asparaginase, and *E*toposide) and 20 sessions of radiation therapy. The right periorbital edema and erythema had almost resolved (see Figure 5). On surveillance imaging, contralateral jugular lymphadenopathy was noted. The patient subsequently had the lymph node biopsy, and the report revealed recurrent NK/T-cell lymphoma. He then underwent 3 cycles of pembrolizumab with complete clinical response. The patient eventually had the matched-unrelated donor peripheral blood stem cell transplant (MUD-PBSCT). It was complicated by severe GI graft-versus-host disease for which the patient was placed on a prolonged course of high-dose steroid therapy. Unfortunately, while on steroid therapy, the patient succumbed to severe *Mycoplasma pneumoniae* pneumonia 2 years after the initial diagnosis of nasal NK/T-cell lymphoma.

## 3. Discussion

Extranodal natural killer/T-cell lymphoma (EKNL) is a rare form of non-Hodgkin's lymphoma and is derived from natural killer (NK) cells or cytotoxic T-lymphocytes [1]. The most common body site of involvement in EKNL is the sinonasal region, and hence it is termed extranodal NK/T-cell lymphoma, nasal type [2]. This type of lymphoma was formerly known as lethal midline granuloma or angiocentric lymphoma [1]. Other organs involvement in EKNL includes salivary glands, skin, breast, gastrointestinal tract, spleen, or testes [2, 3]. The incidence of nasal NK/T-cell lymphoma is highest in Asia (China, Hong Kong, Korea, and Japan) and South America (Peru and Mexico) [1, 2]. It is a rare disease in North America and Europe [2]. Nasal EKNL is virtually always associated with EBV infection [1, 2].

ENKL, nasal type, usually affects the sinonasal region first with extension into the ipsilateral periorbital tissues [2–4]. Clinical manifestations of nasal ENKL include signs and symptoms of recurrent chronic sinusitis, periorbital swelling, chemosis, or proptosis [3, 4]. Rarely, there have been reported cases of periorbital involvement without sinusitis [5]. The diagnosis of nasal ENKL is almost always delayed because of its rarity and oftentimes treated as chronic or acute on chronic sinusitis during initial presentation.

A sinus or orbital tissue biopsy is paramount for arriving at the correct diagnosis. ANCA-associated vasculitis, granulomatosis with polyangiitis, relapsing polychondritis, intranasal cocaine use, and sarcoidosis are important differential diagnoses. Clinical vigilance of nasal ENKL should be raised when there is sinusitis and/or periorbital cellulitis with slow or worsening clinical response to surgical intervention and appropriate antimicrobial therapy.

## 4. Conclusion

Our case illustrates the need to be aware of mimickers of sinusitis/periorbital cellulitis, such as nasal ENKL. Even if cultures are positive, the lack of clinical response or recurrence of sinus/orbital disease despite optimal antimicrobials and surgery should prompt clinicians to consider urgent tissue biopsy in order to rule out other potentially fatal disease processes.

## Data Availability

No data were used to support this study.
